# Evaluation of the anti-spasmodic activity of essential oils of *Ammodaucus leucotrichus* fruits and its main chemical component “perillaldehyde” on intestinal smooth muscle contractions of rodents: *ex vivo* and *in silico* approaches

**DOI:** 10.3389/fchem.2024.1465674

**Published:** 2024-12-23

**Authors:** Ahmed Karim, Mohamed Marghich, Ouafa Amrani, Abdelhay Addous, Sanae Malek, Leila Beyi, Tarik Harit, Dara Aldisi, Mourad A. M. Aboul-Soud, John P. Giesy, Mohammed Aziz

**Affiliations:** ^1^ Laboratory of Bioresources, Biotechnology, Ethnopharmacology and Health, Faculty of Sciences, Mohammed First University, Oujda, Morocco; ^2^ Nutritional Physiopathology, Neurosciences and Toxicology Team, Laboratory of Anthropogenetic, Biotechnology and Health, Faculty of Sciences, Chouaib Doukkali University, El Jadida, Morocco; ^3^ Regional Center for the Professions of Education and Training, Oriental Region, Oujda, Morocco; ^4^ Laboratory of Applied Chemistry and Environment –ECOMP, Faculty of Sciences, Mohammed First University, Oujda, Morocco; ^5^ Department of Community Health Sciences, College of Applied Medical Sciences, King Saud University, Riyadh, Saudi Arabia; ^6^ Department of Clinical Laboratory Sciences, College of Applied Medical Sciences, King Saud University, Riyadh, Saudi Arabia; ^7^ Department of Veterinary Biomedical Sciences and Toxicology Centre, Western College of Veterinary Medicine, University of Saskatchewan, Saskatoon, SK, Canada; ^8^ Department of Integrative Biology and Center for Integrative Toxicology, Michigan State University, East Lansing, MI, United States; ^9^ Department of Environmental Sciences, Baylor University, Waco, TX, United States

**Keywords:** *Ammodaucus leucotrichus*, antispasmodic, essential oil, jejunum, myorelaxant, perillaldehyde

## Abstract

**Ethnopharmacological relevance:**

In Moroccan traditional medicine, plants from the Apiaceae family are widely utilized in folk medicine to treat various diseases associated with the digestive system. *Ammodaucus leucotrichus* plays an important role as an antispasmodic that has been traditionally used, especially to treat digestive tract diseases in children.

**Aim of the study:**

The aim of this research was to verify the traditional use by assessing the relaxant and spasmolytic activities of *A. leucotrichus* essential oil (ALEO) and then comparing them to the effects and potency of the major constituent of ALEO, which is perillaldehyde.

**Materials and methods:**

The *in vitro* evaluation of ALEO’s relaxant and spasmolytic effects was carried out on isolated rats and rabbit jejunum in an organ bath setup. Intestinal contractility was recorded using an isotonic transducer connected to an amplifier. GC/MS analysis was conducted to identify components within ALEO. Subsequently, these compounds underwent *in silico* absorption, toxicity, and molecular docking studies.

**Results:**

GC/MS analysis of this essential oil studied revealed seven compounds, which account for 98.67% of the oil, with the dominance of two compounds, namely, perillaldehyde (91.12%) and limonene (6.33%). ALEO and its main compound, perillaldehyde, reversibly relaxed the basal tone of rabbit jejunum, with the IC_50_ values 158.68 ± 13.89 and 95.03 ± 0.93 μg/mL, respectively. Moreover, ALEO caused a dose-dependent spasmolytic effect on Carbachol (CCh) and KCl provoked jejunum contraction in rats. Furthermore, the decrease in contractions of pre-contracted jejunum by CCh was more pronounced for perillaldehyde compared to ALEO, with an IC_50_ value of 68.59 ± 6.57 μg/mL, which was half compared to that of ALEO. The pre-treatment of the tissue with concentrations ranging from 30 to 100 μg/mL caused a rightward and downward shift in the concentration–response curves for CaCl_2_ and CCh. These results suggest that the spasmolytic effect of ALEO is mediated possibly through a non-competitive antagonist of calcium channel or muscarinic receptors. Our results are confirmed by the fact that perillaldehyde exhibited the highest docking scores on muscarinic acetylcholine receptors (M_2_ and M_3_) and voltage-gated calcium channels, with D-limonene showing lower binding energies in comparison. These remarks confirm that the activity of ALEO is attributed to the presence of perillaldehyde. In addition, perillaldehyde exhibits a low degree of *in silico* acute toxicity and high percent of intestinal absorption.

**Conclusion:**

In summary, ALEO exhibits myorelaxant and antispasmodic effects by inhibiting muscarinic receptors and calcium channels, which can be attributed to the presence of perillaldehyde. This provides a scientific foundation for the traditional use of *A. leucotrichus* in treating gastrointestinal disorders and opens up possibilities for developing a more effective and less toxic drug-utilizing perillaldehyde.

## 1 Introduction

Wooly cumin*, Ammodaucus leucotrichus* Cosson et Durieu (formerly known as *Cuminum maroccanum* P.H. Davis and Hedge), is a small, annual plant from the family “Apiaceae.” It typically grows to 10–20 cm tall and has glabrous, erect, finely striated stems. The leaves are finely dissected and slightly fleshy. The fruit is diachene, 6–10 mm long, ovoid, and densely covered with yellowish-brown soft hair. This plant is found in the Saharan and sub-Saharan regions of North and Tropical Africa and the Canary Islands (Maberly, 1997). *Ammodaucus leucotrichus* Cosson et Durieu is the only species within the genus *Ammodaucus* (Ozenda, 1991), which is known as “Kammun Sofi” in Morocco (Bernard et al., 1997; Maberly, 1997). Fruits of *A. leucotrichus* are widely used as a condiment or spice, as well as in traditional medicine to treat symptoms of common cold, fever, and digestive complaints, particularly in children (Bellakhdar, 2020; Fakchich and Elachouri, 2014). In addition, it has been reported to be used for various biological effects, such as antibacterial, antifungal, and antitumor activities (Abu Zarga et al., 2013; Brahim et al., 2014; Manssouri et al., 2016). Furthermore, essential oil extracted from *A. leucotrichus* has demonstrated potential in treating Alzheimer’s disease. In addition, extracts from *A. leucotrichus* fruits exhibit antioxidant, antihemolytic, and anticoagulant potentials (Laroui et al., 2023). The plant has also been tested for its ability to prevent or cure urinary calculi by inhibiting calcium oxalate crystallization *in vitro* (Beghalia et al., 2009). The essential oil of *A. leucotrichus* exhibited a promising anti-butyrylcholinesterasic activity, with perillaldehyde and limonene having IC_50_ values of 42.7 μg/mL and 66.7 μg/mL, respectively (Sadaoui et al., 2018). This opens the possibility for developing a more effective and less toxic drug-utilizing plant molecules. Many of today’s valuable drugs, including atropine, ephedrine, and digoxin, were developed through the investigation of medicinal plant-based treatments (Aftab and Hakeem, 2021). However, no study on the antispasmodic effect of *A. leucotrichus* essential oil (ALEO) had been performed. Based on its folkloric use as an antispasmodic remedy, this work aims to evaluate the antispasmodic and myorelaxant activities of ALEO in the rodent jejunum. Moreover, an *in silico* predictive study was undertaken to assess the potential toxicity of perillaldehyde, the major compound in ALEO, and explore the correlation between the activities of ALEO obtained and this molecule.

## 2 Materials and methods

### 2.1 Plant material

The wooly cumin, *A. leucotrichus* (Apiaceae), which is known as “Kammun Sofi” in Morocco, was collected in Errachidia city, Morocco (31°55′53″N and −4°25′35″W). Professor Benyounes Haloui from the Faculty of Sciences at Mohammed First University in Morocco assisted in identifying the plant. The specimen labeled HUMPOM4 is stored in the Faculty of Sciences herbarium (Oujda, Morocco). The plant’s identity was verified and validated using http://www.theplantlist.org.

### 2.2 Hydro-distillation of plant material

The essential oil of *A. leucotrichus* (ALEO) was obtained via hydrodistillation; an aliquot of 100 g of *A. leucotrichus* fruits was introduced into a flask, to which 1,000 mL of distilled water was added and boiled for 3 h in the Dean–Stark hydro-distiller. The essential oil was stored at 4°C in amber glass vials until analysis; during the experiment, the essential oil was diluted in dimethyl sulfoxide (DMSO). The yield of the essential oil obtained was calculated as follows (Da Costa et al., 2014):

$$\text{Yield }\text{of }\text{ALEO}=\frac{\text{Weight}\;\text{of}\;\text{the}\;\text{extracted}\;\text{oil}\;\left(g\right)\;}{\text{Weight}\;\text{of}\;\text{plant}\;\text{material}\left(g\right)}.$$

### 2.3 Gas chromatography–mass spectrometry analysis of *A. leucotrichus* fruit essential oil

The essential oil was analyzed using gas chromatography coupled with mass spectrometry (GC/MS), following the methodology described by Beyi et al. (2023). The analysis was conducted in the Chemistry Department of the Faculty of Sciences, Oujda (Morocco).

### 2.4 Solutions and chemicals

The compositions of the solutions employed are as follows: normal Krebs–Henseleit buffer (KHB) solution consisted sodium chloride (118.0 mM), potassium chloride (4.7 mM), calcium chloride (2.5 mM), magnesium sulfate (1.2 mM), sodium bicarbonate (25.0 mM), potassium dihydrogen phosphate (1.2 mM), and glucose (10.0 mM). Calcium-free high K^+^ KHB consisted of sodium chloride (48.0 mM), potassium chloride (75.0 mM), calcium chloride (0.0 mM), magnesium sulfate (1.2 mM), sodium bicarbonate (25.0 mM), potassium dihydrogen phosphate (1.2 mM), and glucose (10.0 mM). Calcium-free KHB consisted of sodium chloride (121.7 mM), potassium chloride (4.7 mM), calcium chloride (0.0 mM) magnesium sulfate (1.2 mM), sodium bicarbonate (25.0 mM), potassium dihydrogen phosphate (1.2 mM), and glucose (10.0 mM).

All solutions were prepared using distilled water, and the pH was adjusted to 7.4. Drugs utilized in the study included atropine sulfate, verapamil hydrochloride, carbamylcholine chloride (carbachol; CCh), NaCl, H_2_PO4, MgSO_4_, CaCl_2_, NaHCO_3_, KCl, and glucose; all these drugs are obtained from Sigma Chemical Co. (Sigma-Aldrich, United States). Perillaldehyde was obtained from Thermo Scientific, and DMSO was purchased from Prolabo. All these chemicals used were of analytical quality.

### 2.5 Animals

The animals used in this study were as follows:

*Wistar* rats (150–300 g) and *New Zealand* rabbits (1.5–2 kg) of both sexes were accommodated in the animal facilities of the Faculty of Sciences, Oujda, Morocco. They had access to water and standard food and held on a light–dark cycle (12 h/12 h). Animals were allowed to fast for 18 h prior to the experiments. All animal-related procedures adhered to ethical standards outlined in the Guide for the Care and Use of Laboratory Animals by the National Research Council (NRC, 2011). The study protocol received approval from the Moroccan Ethics Committee for Animal Research (MECAR) under the reference number UCD-FSJ-06/2024.

### 2.6 Rodent intestinal smooth muscle preparation

Rats and rabbits were anesthetized with ethyl ether inhalation, and their jejunum were removed and incubated in oxygenated normal KHB for 60 min at 37°C with a pH of 7.4. A 2-cm jejunum segment was immersed in organ baths containing the normal KHB solution and changed every 15 min for equilibration. The impact of each dosage was documented for a minimum of 7 min. Effective jejunum contraction with a potassium-rich medium (KCl 75 mM) was ensured. Jejunum contractions were documented using an isotonic transducer connected to an amplifier and data acquisition software PROTOWIN Panlab.

### 2.7 Relaxant study of ALEO and perillaldehyde on the basic contraction of jejunum of rabbits

After stabilizing the basic contractions of jejunum of rabbits, ALEO and perillaldehyde (from 10 to 300 μg/mL) were added to the organ bath. Verapamil (0.1, 0.3, and 1 μM) served as the positive control.

### 2.8 Antispasmodic effect of ALEO and Perillaldehyde on the rat jejunum contracted using potassium chloride and carbachol

After stabilizing jejunal contractions, the smooth muscle was contracted using 25 mM potassium chloride (KCl) or CCh (10^−6^ M). Then, after the stabilization of the contractions, cumulative doses of ALEO or perillaldehyde were added to the organ bath (from 10 to 150 μg/mL).

### 2.9 Spasmolytic activity of ALEO on dose–response curves of carbachol

Dose–response curves for carbachol were generated for the jejunum using the van Rossum procedure (Rossum, 1963). Following the equilibration period of 30 min, CCh from 10^−8^ to 10^−5^ M was introduced into the isolated jejunum bath under two conditions: without any additions (control) and in the presence of atropine (10^−6^ M) or various doses of ALEO (30, 50, and 100 μg/mL).

### 2.10 Inhibition of dose–response of calcium chloride by ALEO

After 30 min of equilibration in the normal Krebs–Henseleit buffer (KHB) solution, this latter was replaced with calcium-free KHB for 15 min, followed calcium-free high K^+^ KHB. Cumulative dose–response curves to calcium chloride ranging from 0.1 to 10 mM were generated under two conditions: without any additions (control) and in the presence of varying doses of ALEO (30, 50, and 100 μg/mL) or verapamil (10^−6^ M).

### 2.11 Docking study

The docking calculations were carried out using the freely available “iGEMDOCK program” (Yang and Chen, 2004). The human M_2_ muscarinic acetylcholine receptor (3UON), the M_3_ muscarinic acetylcholine receptor (4DAJ), and the rabbit Cav1.1-verapamil complex complexed with verapamil (6JPA) were retrieved from the Protein Data Bank (http://www.rcsb.org./pdb). Perillaldehyde in the three-dimensional form was obtained from the National Library of Medicine (https://pubchem.ncbi.nlm.nih.gov/compound/Perillaldehyde). A 3D structure of D-limonene (ZINC968226) was obtained from the ZINC database (https://zinc.docking.org/substances/ZINC000000968226/). The drug screening parameter was employed during the calculations. Both 3D and 2D docked poses were obtained via RasMol 2.7.3 (www.rasmol.org) and BIOVIA Discovery Studio Visualizer 2021 v21.1.0.20298 (https://discover.3ds.com/discovery-studio-visualizer-download), respectively.

### 2.12 *In silico* prediction of absorption properties and toxicity of perillaldehyde

Absorption profiles were evaluated *in silico* using the computational tool pkCSM web server (http://biosig.unimelb.edu.au/pkcsm/, accessed on 31 December 2023) (Pires et al., 2015). The assessment of LD_50_ values, toxicity class, hepatotoxicity, carcinogenicity, immunotoxicity, mutagenicity, and cytotoxicity was conducted utilizing the ProTox II online tool (https://tox-new.charite.de/protox_II/, accessed on 31 December 2023) (Banerjee et al., 2018).

### 2.13 Statistical analyses

Normality was checked using the Shapiro–Wilk test, and the assumption of homogeneity of variance was evaluated using Levene’s test. The results of the tests carried out are expressed as the mean ± SEM. The difference between the results of essential oils and the controls was performed by a one-way analysis of variance (ANOVA), followed by Bonferroni’s multiple comparison tests. Concentration–response curves were graphed, and nonlinear regression analysis was performed using the Hill equation through an iterative least-squares method (GraphPad Prism 5.0 Software for Windows, San Diego, CA, United States). This approach aimed to derive estimates of effective concentration 50 (EC_50_), which is the concentration of the agonist that causes half-maximal contraction in the presence of the antagonist. Differences are considered statistically significant when the probability value *p* is less than 5% [(*): *p* < 0.05, (**): *p* < 0.01, and (***): *p* < 0.001].

## 3 Results

### 3.1 Yield of ALEO

The yield of ALEO obtained following hydro-distillation was 2.17%.

### 3.2 Gas chromatography of ALEO

**FIGURE 1:**
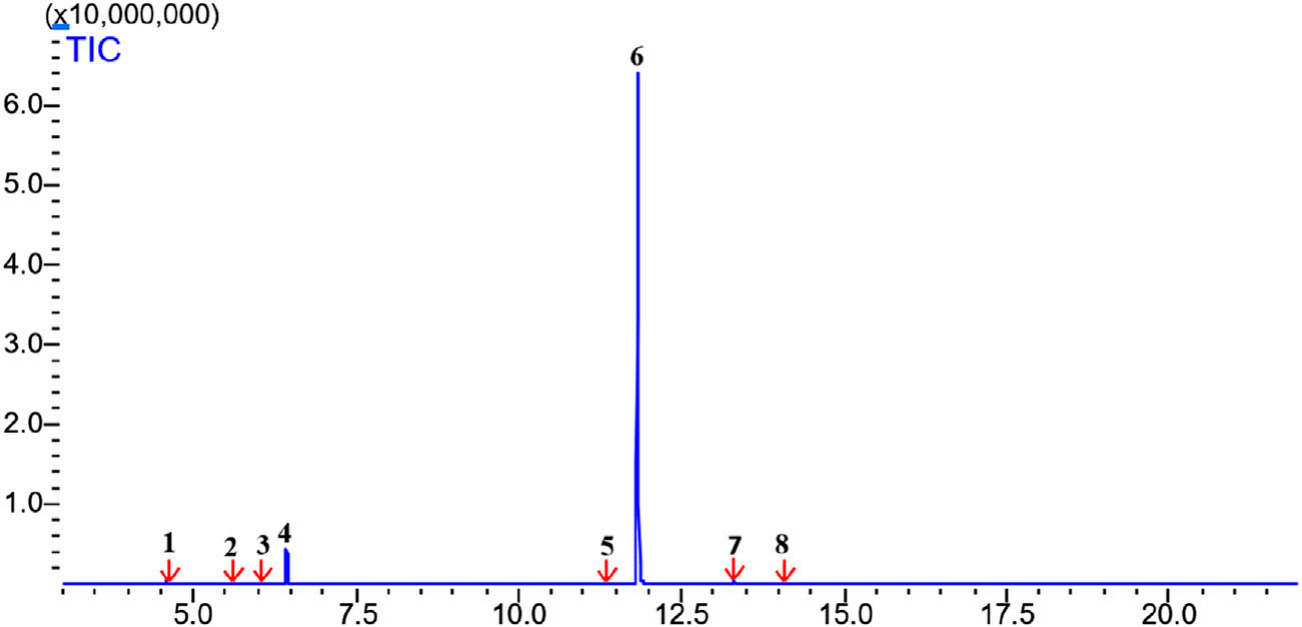
Chromatogram from GC/MS analysis of ALEO: 1: alpha-pinene; 2: beta-pinene; 3: 3-carene; 4: D-limonene; 5: 3-isopropylbenzaldehyde; 6: perillaldehyde; 7: methyl (2E)-(2isopropenylcyclopentylidene)ethanoate; 8: eudesma-4 (14), 11-diene.

**FIGURE 2:**
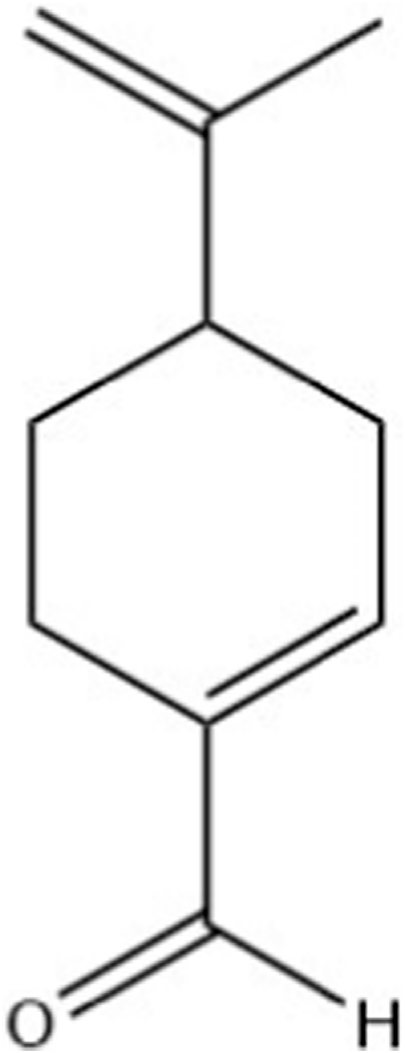
Chemical structure of perillaldehyde.

**FIGURE 3:**
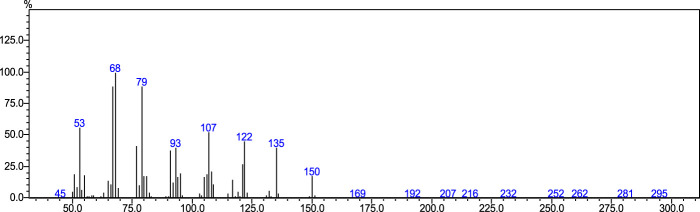
Mass spectrum obtained from GC/MS analysis of perillaldehyde.

Perillaldehyde was the major component found in ALEO with the highest percentage equal to 91.12%, followed by D-limonene by 6.33% (Table 1; Figure 1). The molecular structure and mass spectrum of perillaldehyde, as determined through GC-MS analysis, are illustrated in Figures 2 and 3.

**TABLE 1:** Chemical composition of the ALEO fruit.

N°	Component	Retention time	Percentage (%)
1	Alpha-Pinene	4.580	0.52
2	Beta-Pinene	5.559	0.22
3	3-Carene	5.994	0.24
4	D-Limonene	6.436	6.33
5	3-Isopropylbenzaldehyde	11.306	0.07
6	Perillaldehyde	**11.840**	**91.12**
7	Methyl (2E)-(2-isopropenylcyclopentylidene)ethanoate	13.310	0.90
8	Eudesma-4 (14),11-diene	13.866	0.17
	Total		**99.57**

The bolded values emphasize the retention time and percentage of perillaldehyde, the principal compound in ALEO.

### 3.3 Myorelaxant study of ALEO and perillaldehyde on the spontaneous contraction of the rabbit jejunum

**FIGURE 4:**
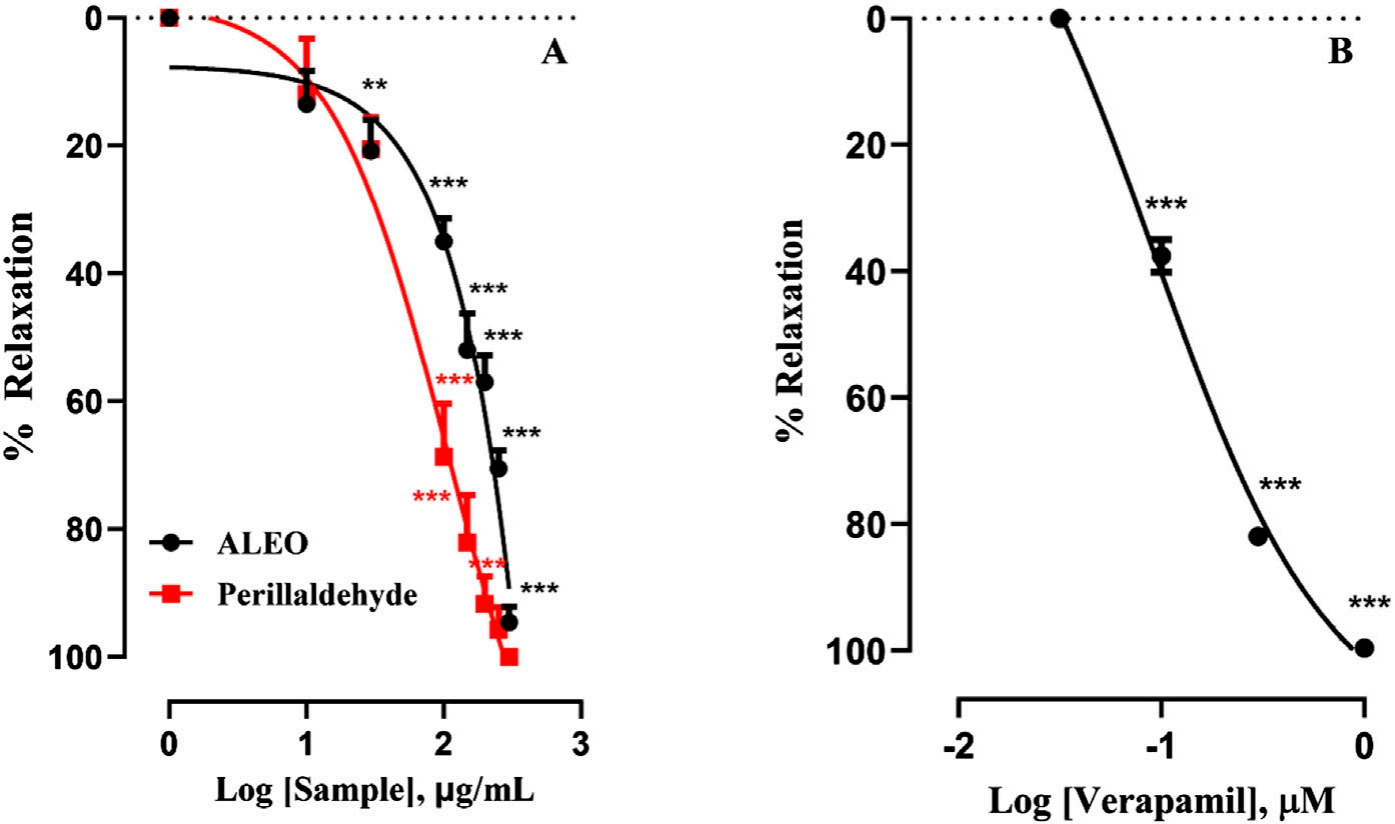
Effect of *A. leucotrichus* essential oil (ALEO), perillaldehyde **(A)**, and verapamil **(B)** on the basal tone of the rabbit jejunum. **, *p* < 0.01 and ***, *p* < 0.001. The disparity exhibits statistical significance within the control group (the zero line). Mean ± S.E.M; n = 5.

**FIGURE 5:**
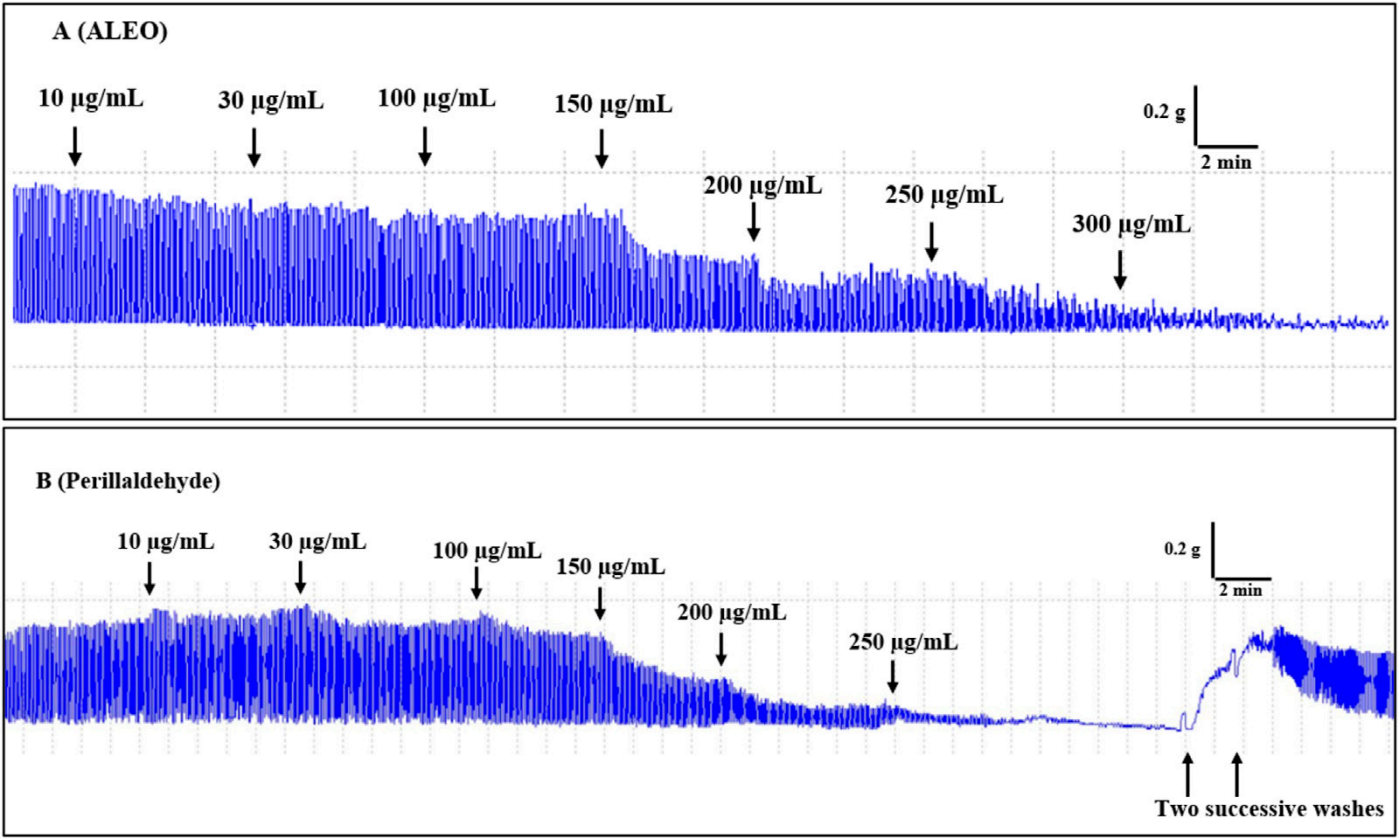
Original records of the myorelaxant impact of the *A. leucotrichus* essential oil (ALEO) **(A)** and perillaldehyde **(B)** on the basic jejunum rabbit contractions.

Cumulative doses ranging from 10 to 300 µg ALEO/mL resulted in a dose-dependent relaxation of rabbit jejunum basal contractions (Figure 4A), with an IC_50_ value of 158.68 ± 13.89 μg/mL (Table 2). The maximum effect of ALEO was observed at 300 μg/mL, and the contractions resume after washing with physiological liquid (Figure 5A). Perillaldehyde, the primary component of the ALEO, exhibited a similar inhibitory effect on baseline contractions in the rabbit jejunum, with a maximum inhibitory effect at 250 μg/mL, as shown in Figure 5B, and a lesser IC_50_ value (95.03 ± 0.93 μg/mL) compared to whole ALEO (Table 2). These results were compared to those of verapamil, serving as a positive control and employed as a calcium channel antagonist (Figure 4B), with an IC_50_ value of 44.19 ± 0.001 μg/mL (Table 2).

**TABLE 2:** IC_50_ values for relaxant effects of *A. leucotrichus* essential oil (ALEO), perillaldehyde, and verapamil on rabbit basic contractions of jejunal smooth muscle.

Sample	ALEO (μg/mL)	Perillaldehyde (μg/mL)	Verapamil (μg/mL)
IC_50_	158.68 ± 13.89	95.03 ± 0.93	44.19 ± 0.001

IC_50_: The concentration of the sample inhibits 50% of the rabbit basal contractions.

### 3.4 Spasmolytic activity of ALEO and perillaldehyde on the rat jejunum contracted by CCh and KCl

**FIGURE 6:**
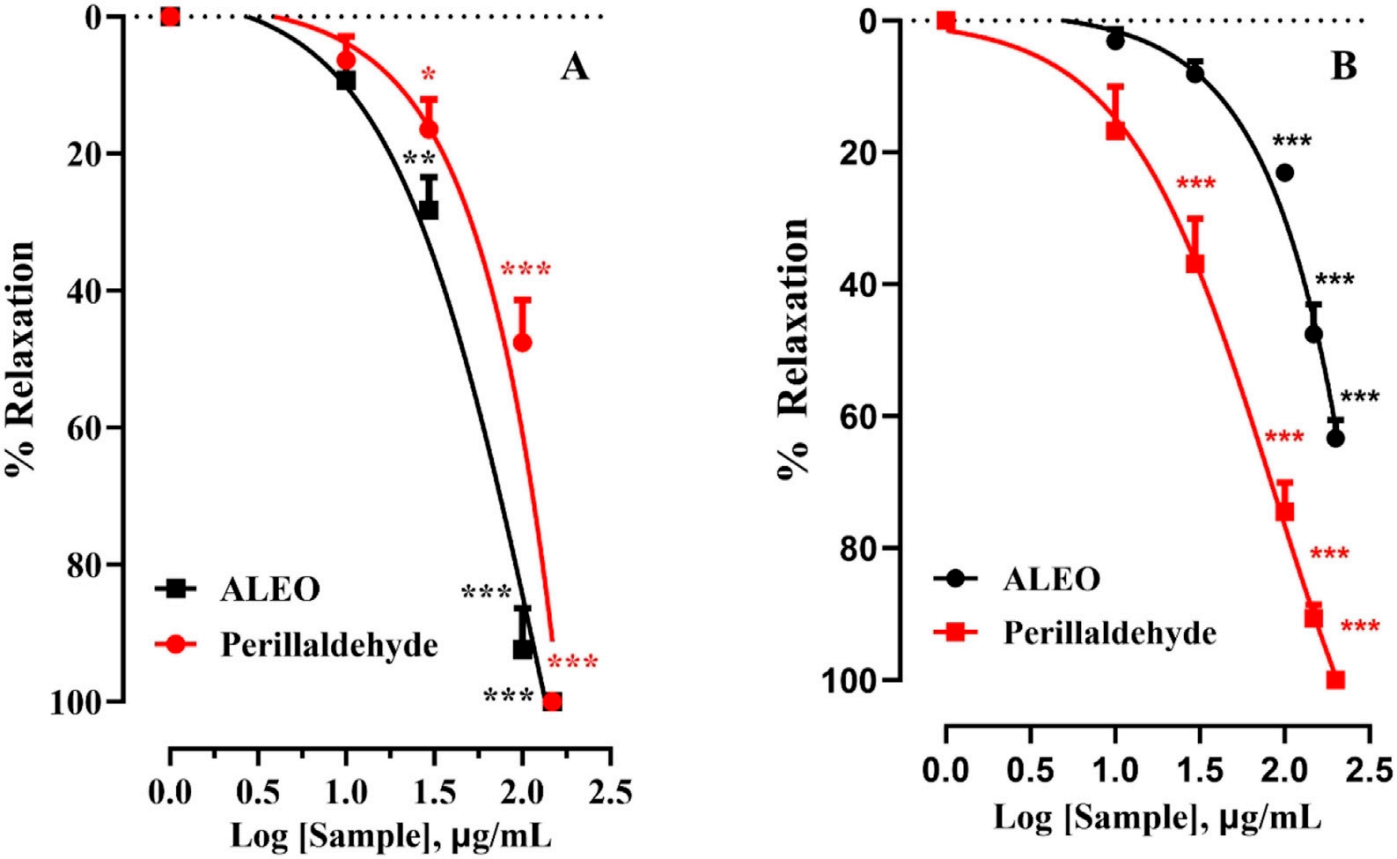
*A. leucotrichus* essential oil (ALEO) and perillaldehyde effects on the jejunum contracted by KCl (25 mM) **(A)** and CCh (10^−6^ M) **(B)**. **, *p* < 0.01 and ***, *p* < 0.001. The disparity exhibits statistical significance within the control group (the zero line). Mean ± S.E.M; n = 5.

The effect of ALEO on the rat jejunum contracted by carbachol was observed (Figure 6). The IC_50_ value for ALEO was 172.63 ± 14.8 μg/mL (Table 3). ALEO caused a dose-dependent spasmolytic effect on CCh-induced rat jejunal contraction (Figure 6B). The decrease in contractions of the pre-contracted jejunum by CCh was more pronounced for perillaldehyde compared to ALEO (Figure 6B), with an IC_50_ value of 68.59 ± 6.57 μg/mL, which was half of the IC_50_ value for ALEO (Table 3).

**TABLE 3:** IC_50_ values for the antispasmodic activity of *A. leucotrichus* essential oil (ALEO) and perillaldehyde on the rat jejunum pre-contracted by CCh or KCl.

	IC_50_ (µg/mL)	
	KCl (25 mM)	CCh (10^−6^ M)
ALEO	87.63 ± 6.65	172.63 ± 14.8
Perillaldehyde	89.48 ± 9.30	68.59 ± 6.57

IC_50_: The concentration of ALEO that inhibits 50% of the smooth muscle pre-contracted by KCl or CCh.

The impact of ALEO on the rat jejunum contracted by potassium chloride is depicted in Figure 6A. The obtained IC_50_ value for ALEO is 87.63 ± 6.65 μg/mL, as detailed in Table 3. Notably, ALEO demonstrated a dose-dependent antispasmodic effect on KCl-induced contractions in the rat jejunum (Figure 6A). In addition, the reduction in contractions of the pre-contracted jejunum by KCl was comparable to perillaldehyde and ALEO (Figure 6A), with an IC_50_ value of 89.48 ± 9.30 μg/mL (Table 3).

### 3.5 Spasmolytic activity of ALEO on dose–response curves of carbachol on the rat jejunum

**FIGURE 7:**
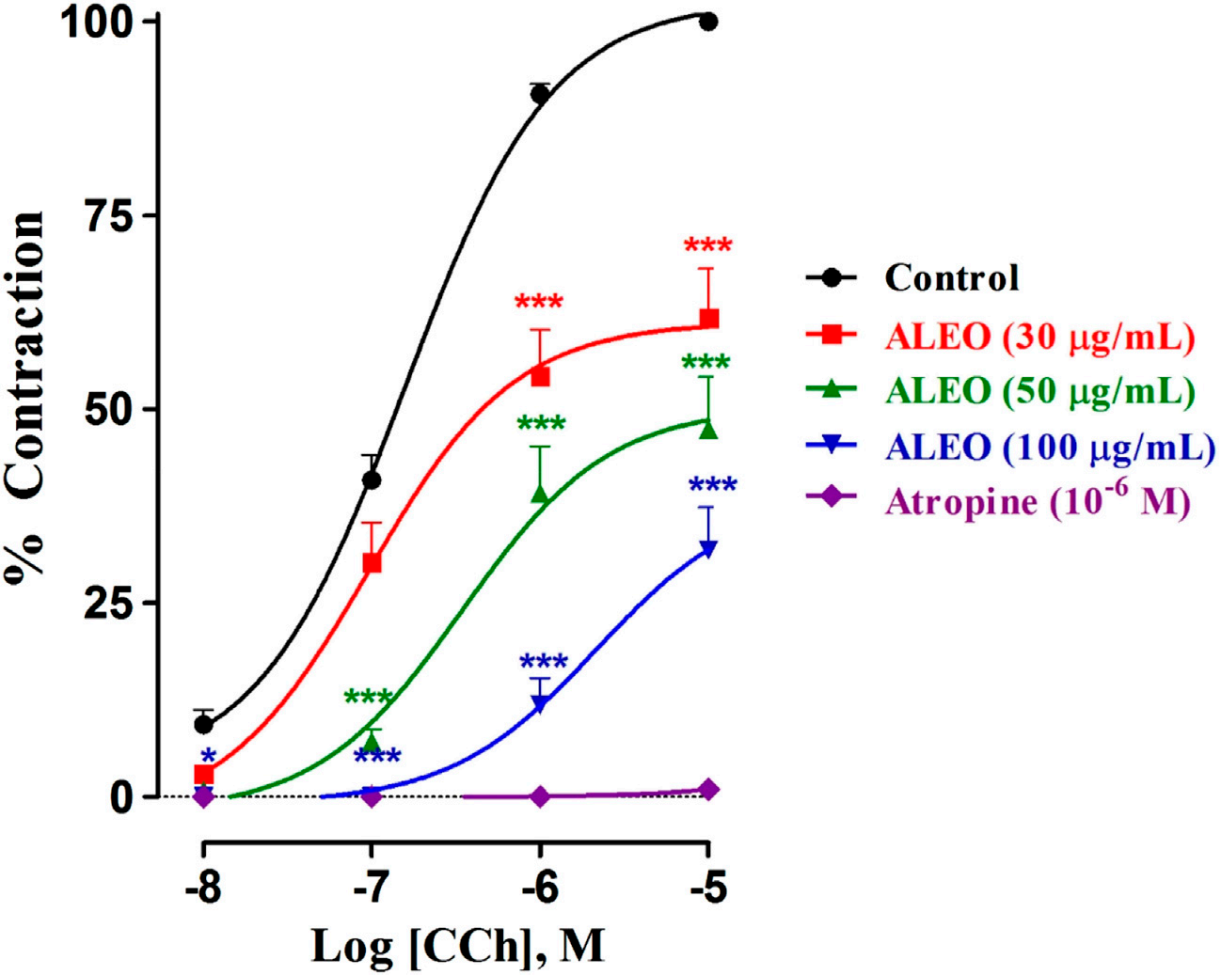
Dose–response curves of carbachol on the rat jejunum in the absence and presence of various concentrations of *A. leucotrichus* essential oil (ALEO) or atropine (10^−6^ M). *, *p* < 0.05 and ***, *p* < 0.001. The disparity exhibits statistical significance within the control group. Mean ± SEM, n = 6.

The findings of the investigation, reveal that varying concentrations of ALEO (30, 50, and 100 μg/mL) effectively mitigated the heightened tone induced by cumulative CCh doses (Figure 7). This mitigation was evident in the downward and rightward shifts of dose–response curves. Atropine served as a positive control. The effective concentration of the agonist (CCh) that inhibited 50% of maximal contraction in the absence of the antagonist was 1.58 × 10^−7^ M. Conversely, exposure to 100 μg of ALEO/mL had an EC_50_ value of 2.16 × 10^−6^ M. This suggests that the concentration needed for a 50% contraction was greater in the presence of ALEO (Table 4).

**TABLE 4:** Half-maximal effective concentration value (EC_50_) determined from CCh dose–response curves on the rat jejunum in the absence and presence of various doses of *A. leucotrichus essential oil* (ALEO) or atropine (10^−6^ M).

	Control	ALEO (30 μg/mL)	ALEO (50 µg/mL)	ALEO (100 μg/mL)	Atropine (10^−6^ M)
EC_50_ (M)	1.58 × 10^−7^	9.63 × 10^−8^	3.46 × 10^−7^	2.16 × 10^−6^	0.072

EC_50_: The effective concentration of the agonist (CCh) that gives 50% of maximal contraction in the presence of the antagonist (ALEO or atropine).

### 3.6 Dose–response for the inhibition of calcium chloride by ALEO on the rat jejunum

**FIGURE 8:**
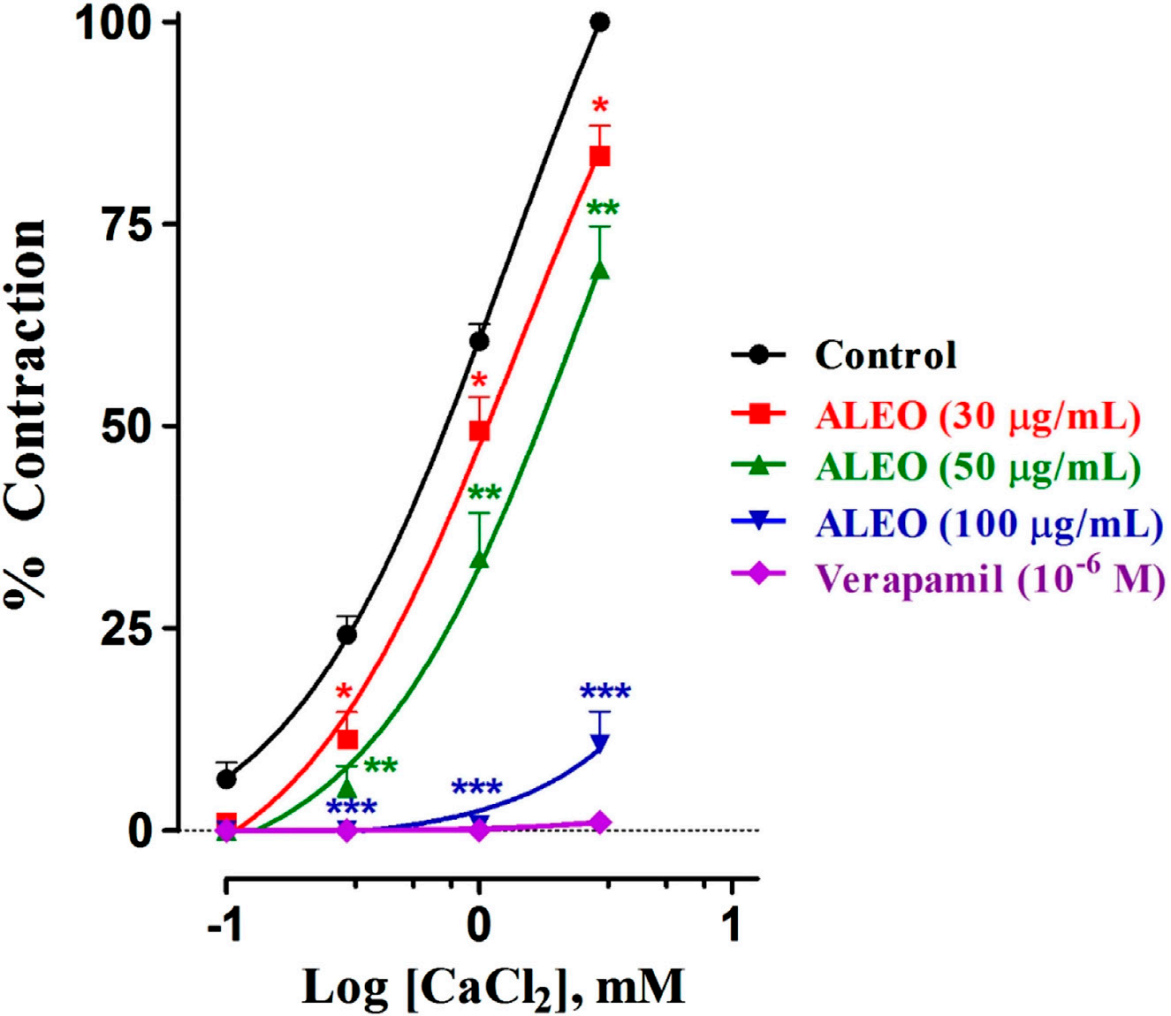
Dose–response curves of CaCl_2_ on the rat jejunum in the addition or the absence of various doses of *A. leucotrichus* essential oil (ALEO) or verapamil (10^−6^ M). *, *p* < 0.05; **, *p* < 0.01; and ***, *p* < 0.001. The disparity exhibits statistical significance within the control group. Mean ± SEM; n = 6.

ALEO inhibited cumulative doses of CaCl_2_ (Figure 8). This inhibition is evident through the downward and rightward shifts observed in the dose–response curves. Notably, the dose of 100 μg/mL demonstrated particularly effective inhibition. Verapamil served as the positive control in the study. The log of EC_50_ for CaCl_2_ in the control group was 0.114 ± 0.016 mM. Conversely, 100 μg of ALEO/mL increased the log of EC_50_ value significantly to 4.7 ± 0.001 mM. This substantial increase indicates that the concentration required to trigger a 50% contraction by CaCl_2_ was greater in the presence of ALEO (Table 5).

**TABLE 5:** Log half-maximal effective concentration values obtained from calcium chloride dose–response curves on the rat jejunum in the presence or absence of various doses of *A. leucotrichus essential oil* (ALEO) or verapamil (10^−6^ M).

	Control	Verapamil (10^−6^ M)	ALEO (30 μg/mL)	ALEO (50 μg/mL)	ALEO (100 μg/mL)
Log EC_50_ (mM)	0.114	4.681	0.142	0.430	4.679
SEM	0.016	0.001	0.163	0.191	0.001

EC_50_ (half-maximal effective concentration): concentration of the agonist (CaCl_2_) that gives 50% of maximal contraction in the presence of the antagonist (ALEO or verapamil).

### 3.7 Docking study

**FIGURE 9:**
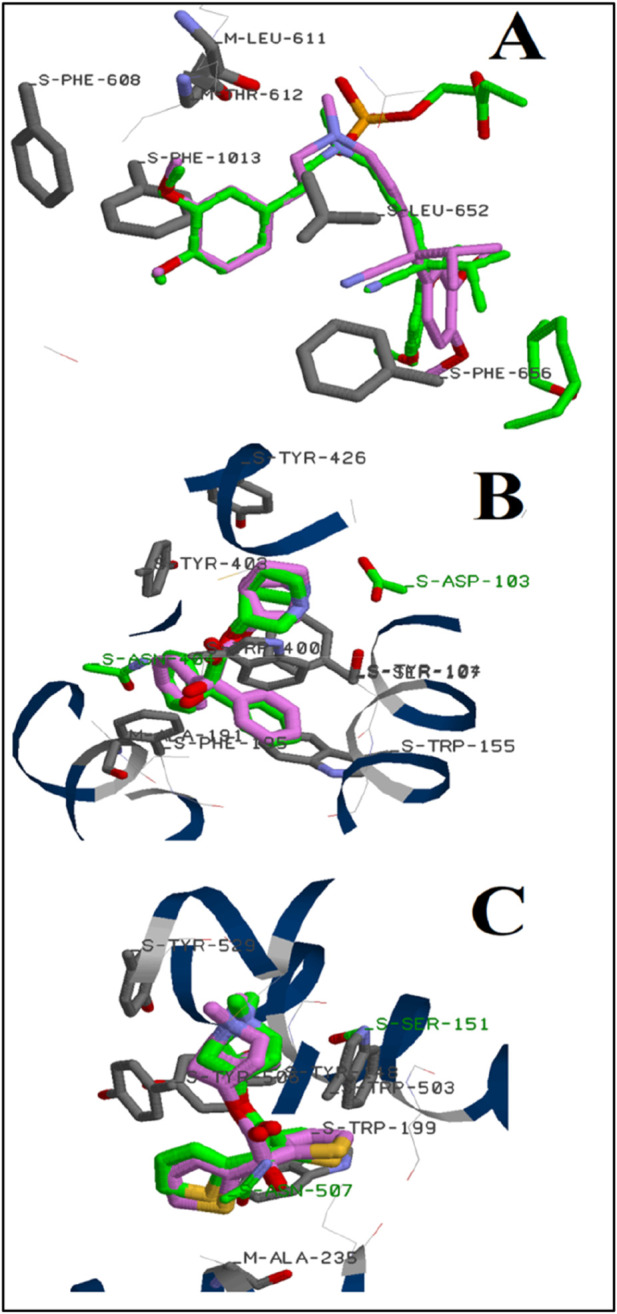
Position of verapamil **(A)**, 3-quinuclidinyl benzilate **(B),** and tiotropium **(C)** on the active site of L-type voltage-gated calcium (Ca_v_1.1) channels, human M_2_ muscarinic acetylcholine receptor, and the M_3_ muscarinic acetylcholine receptor, respectively: (green color); (pink color) the predicted docking poses.

The principal component of ALEO was perillaldehyde, which constituted 91.12% of the mass. To compare with the experimental findings, this compound was docked with the L-type voltage-gated calcium (Ca_v_1.1) channel active sites (6JPA), as well as the human M_2_ muscarinic acetylcholine receptor (3UON) and the M_3_ muscarinic acetylcholine receptor (4DAJ). The validation technique used during the calculation was established in our previous study (Marghich et al., 2023), and the results displayed in Figure 9 evidenced that the docked ligands (verapamil, 3-quinuclidinyl benzilate, and tiotropium) and ligands in the corresponding complexes occupied the same confirmations via the same interactions.

**FIGURE 10:**
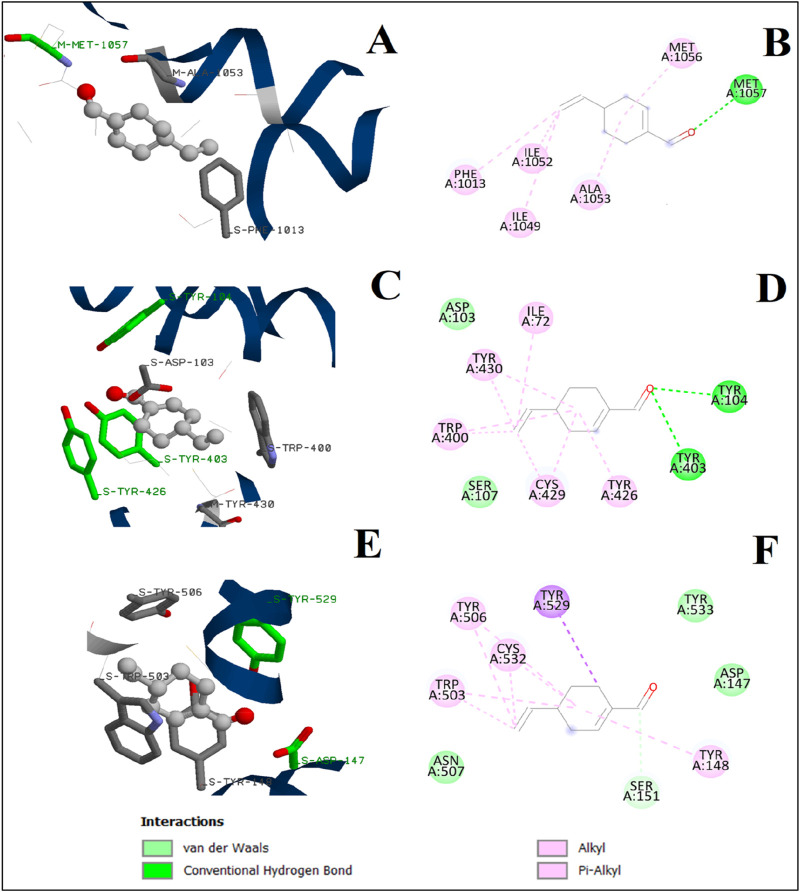
3D and 2D interactions of perillaldehyde with L-type voltage-gated calcium (Ca_v_1.1) channel active site **(A, B)**, human M_2_ muscarinic acetylcholine receptor **(C, D)**, and M_3_ muscarinic acetylcholine receptor **(E, F)**.

Perillaldehyde was subjected to the docking calculations under the same docking protocol. Both two- and three-dimensional binding configurations of the studied substances are presented in Figure 10. For the Ca_v_1.1 channel, perillaldehyde forms a conventional hydrogen bond with the oxygen atom and the residue MET1057. Moreover, other residues such as ALA1053 and PHE1063 participate in the stabilization of the complex via alkyl interactions with the cyclohexene moiety. In the case of the human M_2_ muscarinic acetylcholine receptor, the oxygen atom of perillaldehyde is also attached to the active site of the receptor via two conventional hydrogen bonds with TYR104 and TYR403. Furthermore, we observe the existence of two van der Waals interactions, with ASP103 and SER107. By contrast, no conventional hydrogen bonds were observed in the case of the M_3_ muscarinic acetylcholine receptor. Only, van der Waals and π–alkyl interactions were remarked. Finally, the binding energy of perillaldehyde with the active site of these three receptors increases in this order: Ca_v_1.1 channel < M_3_ muscarinic acetylcholine receptor ≈ human M_2_ muscarinic acetylcholine receptor (Tables 6, 7).

**TABLE 6:** Binding energies of perillaldehyde, D-limonene, and atropine with the active sites of the M_3_ muscarinic acetylcholine receptor and the human M_2_ muscarinic acetylcholine receptor.

	Binding energy (Kcal/mol)	
Compound	Human M_2_ muscarinic acetylcholine receptor	M_3_ muscarinic acetylcholine receptor
Perillaldehyde	−61.9	−60.6
D-Limonene	−55.4	−55.2
Atropine	−110.4	−109.6

**TABLE 7:** Binding energies of perillaldehyde, D-limonene, and verapamil with the active sites of the L-type voltage-gated Ca^2+^ (Ca_v_1) channel.

Compound	Binding energy (Kcal/mol)
Perillaldehyde	−49.8
D-Limonene	−44.7
Verapamil	−108.27

**FIGURE 11:**
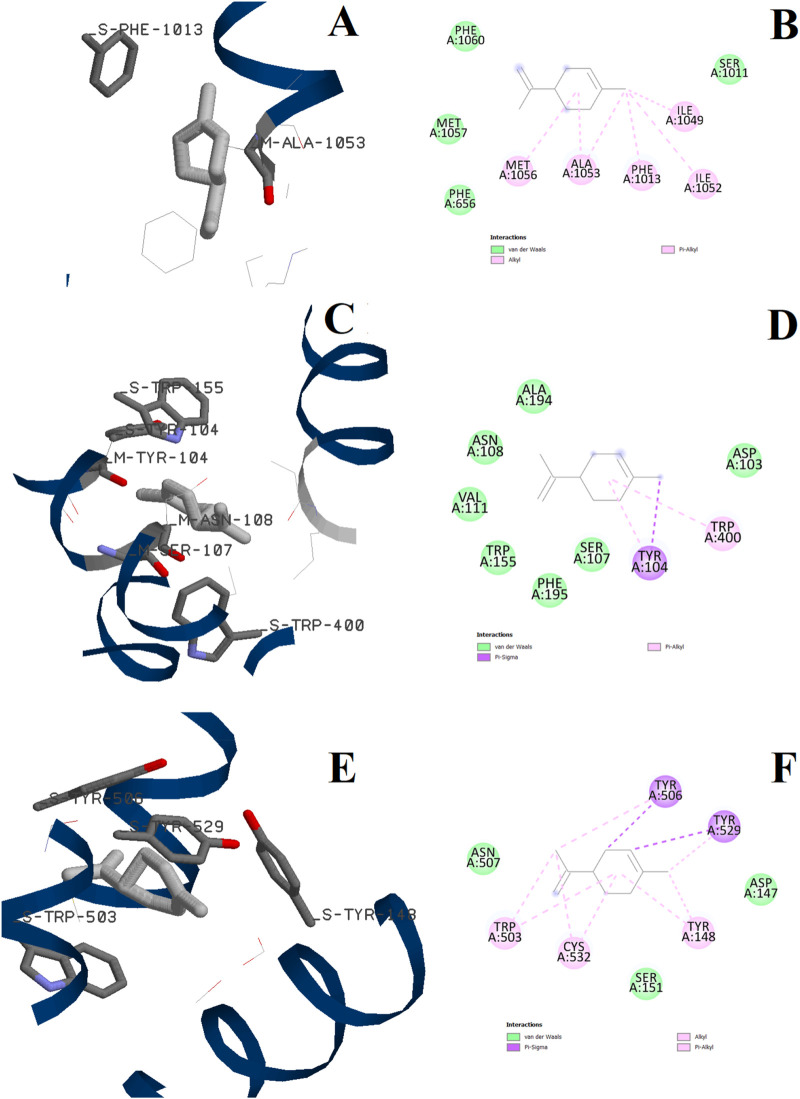
3D and 2D interactions of D-limonene with the L-type voltage-gated Ca^2+^ (Ca_v_1) channel active site **(A, B)**, the human M2 muscarinic acetylcholine receptor **(C, D)**, and the M3 muscarinic acetylcholine receptor **(E, F)**.

Docking calculations were also performed for D-limonene as it is present in the chemical composition of ALEO with the percentage of 6.33%. The 3D and 2D binding configurations are displayed in Figure 11, while the binding energies are represented in Tables 6, 7. We note that the D-limonene performs some interactions with the active sites of the three targets. However, its binding energies were found less than those of perillaldehyde. In addition, the percentage of perillaldehyde (91.2%) is higher than that of D-limonene (6.33%). These remarks confirm that the activity of ALEO is associated with the presence of perillaldehyde more than D-limonene.

### 3.8 *In silico* prediction of absorption properties and toxicity of perillaldehyde

Perillaldehyde exhibits several unique pharmacokinetic properties associated with absorption (Table 8). These include, among others, low water solubility with a logarithmic value of −2.482 mol/L. This suggests that perillaldehyde is more likely to dissolve in lipids than in aqueous solutions. Relative to absorption by the human intestine, a large percentage (97.2%) of perillaldehyde is absorbed in the human intestinal tract, which suggests efficient absorption from the gut. In addition, the Caco-2 permeability assay, which measures the ability of a substance to pass through the intestinal epithelium (Hubatsch et al., 2007), indicates that perillaldehyde has a log Papp value higher than 0.9. This suggests that they have the greatest likelihood of being absorbed through the intestinal wall. In addition, perillaldehyde has low permeability through the skin. In addition, it can bind to the P-glycoprotein transporter.

**TABLE 8:** *In silico* absorption prediction of perillaldehyde.

	Unit	Perillaldehyde
Water solubility	Log mol/L	−2.482
CaCo_2_ permeability	Log Papp in 10^−6^ cm/s	1.507
Intestinal absorption (human)	% absorbed	97.185
Skin permeability	log Kp	−2.727
P-glycoprotein substrate	Categorical (yes/no)	Yes
P-glycoprotein I inhibitor		No
P-glycoprotein II inhibitor		No

Table 9 shows that perillaldehyde has an LD_50_ value of 1720 mg/kg of body weight, indicating a low level of acute toxicity. This compound is classified as a class 4 toxicant, signifying a moderate degree of acute toxicity, whereas a higher-class number generally correlates with reduced toxicity severity. Notably, Perilladehyde is not expected to induce hepatotoxicity, carcinogenicity, immunotoxicity, mutagenicity, or cytotoxicity.

**TABLE 9:** *In Silico* toxicity prediction of perillaldehyde.

	Perillaldehyde
LD_50_ mg/Kg	1720 mg/Kg
Class	4
Hepatotoxicity	Inactive
Carcinogenicity	Inactive
Immunotoxicity	Inactive
Mutagenicity	Inactive
Cytotoxicity	Inactive

## 4 Discussion

In Morocco, plants from the Apiaceae family are widely utilized in folk medicine to treat various diseases associated with the digestive system (Es-Safi et al., 2020; Redouan et al., 2020). *A. leucotrichus* Coss. and Dur. (hairy cumin) holds significance in traditional medicine across North African countries, with a particular emphasis on its use in Morocco. Fruits of this plant are employed in the treatment of various ailments, including gastralgia, indigestion, and gastrointestinal pain. In addition, they are commonly utilized to address digestive tract issues in infants, including dysentery, nausea, vomiting, and regurgitation (Hajib et al., 2023; Ziani et al., 2019). Several studies have investigated the essential oils of *A. leucotrichus* for their antimicrobial and antioxidant properties, while additional research has focused on their anti-inflammatory, anti-tumor, and anticholinesterase (Alzheimer’s disease) activities (Abu Zarga et al., 2013; Naima et al., 2019; Rani et al., 2022; Sadaoui et al., 2018). These wide pharmacological effects are probably due to the specific molecular profile of *A. leucotrichus* essential oil (ALEO), which are characterized by the dominance of perillaldehyde as the primary component, with limonene following as a significant constituent with a varying percentage. In the study by Louail et al. (2016), the content was reported as 59.13% for perillaldehyde and 23.89% for limonene. Conversely, Brahim et al. (2014) observed a composition of 88.78% for perillaldehyde and 8.26% for limonene. In addition, Halla et al., 2018 found that ALEO is composed of 85.6% of perillaldehyde, with limonene accounting for 7.8%. In our results, we also found that perillaldehyde is the major component in ALEO with the highest percentage equal to 91.12%, followed by D-limonene by 6.33%. The quantitative difference in chemicals may be ascribed to factors such as harvest time, plant origin, climatic conditions, seasonal variations, and the stage of the vegetative cycle.

The antispasmodic activity of this essential oil has not yet been studied. Therefore, the aims of this investigation were to assess the myorelaxant and antispasmodic effects of ALEO and compare them with the major constituents of ALEO which is perillaldehyde. The results demonstrated a significant dose-dependent relaxation of contractions of the basal jejunum in rabbits that was reversible, with the IC_50_ values of 158.68 ± 13.89 and 95.03 ± 0.93 μg/mL for ALEO and perillaldehyde, respectively. This means that perillaldehyde, the primary contributor to the myorelaxant effect obtained by ALEO, demonstrates higher potency against basal jejunum rabbit contractions than ALEO itself. The IC_50_ value of ALEO is extremely lower than that of LD_50_ (520–570 mg/kg). Mohammedi et al. (2018) affirmed that the doses chosen are non-toxic and effective for antispasmodic activity. Other essential oil herbal medicines like *Origanum majorana* (Makrane et al., 2019), *Thymus algeriensis* (Beyi et al., 2023), and *Warionia saharae* (Amrani et al., 2022) were also more potent than ALEO on spontaneous contraction. These rhythmic and spontaneous contractions of smooth muscle cells (SMCs) are generated by interstitial Cajal cells (ICCs) that create slow waves, conducting them into adjacent SMCs to initiate a depolarization/repolarization cycle (Sanders et al., 1999; Takaki, 2003). ICCs are distinctive cells responsible for generating electrical pacemaker activity in gastrointestinal smooth muscles (Xu, 2011). Therefore, the observed myorelaxant effect serves as evidence that the essential oil or perillaldehyde influences the intrinsic nervous system of the jejunum, specifically targeting the region characterized by inherent automaticity. This finding highlights the fact that the effects of either ALEO or perillaldehyde on the autonomous regulation of contractions of the jejunum, which provides insights into potential interactions with the intricate neural pathways responsible for coordinating rhythmic and spontaneous movements within this particular segment of the intestine. It can therefore be concluded that ALEO and its major component induced relaxation of the jejunum muscle by directly affecting the SMCs and/or on the ICCs. This hypothesis is consistent with the mechanism proposed previously for the essential oil of *Artemisia campestris* as the myorelaxant agent (Marghich et al., 2023).

The mechanisms of contractions of SMCs are closely linked to Ca^2+^ ions. The main source of Ca^2+^ ions responsible for initiating contractions is derived from the external solution and is facilitated by voltage-dependent calcium channels. These latter are primarily influenced by K^+^ and non-selective cation conductances on the electrical potential of the cell membrane and excitability. Additionally, the entry of Ca^2+^ is complemented by the release of Ca^2+^ from inositol 1,4,5-trisphosphate (IP_3_) receptor-operated stores, along with mechanisms that modify the sensitivity of the contractile apparatus to fluctuations in cytoplasmic calcium (Sanders, 2008). To better understand the inhibitory effect of ALEO and perillaldehyde on the contraction of the jejunum via the calcic pathways, the rat jejunum was pre-contracted using potassium chloride (KCl). This causes a depolarization of SMC membranes, inducing the opening of voltage-gated calcium channels and thus increasing intracellular Ca^2+^, ultimately leading to the contraction of intestinal SMCs (Karaki et al., 1984). ALEO exhibited a dose-dependent spasmolytic activity effect on potassium chloride (KCl)-induced contractions on the jejunum in the rat. Additionally, the reduction in contractions of the pre-contracted jejunum by KCl was comparable for perillaldehyde to ALEO. ALEO and perillaldehyde can, therefore, have a blocking action on the opening of voltage-gated calcium channels. This hypothesis was confirmed by the fact that various doses of ALEO effectively inhibited the increase in tone induced by cumulative doses of CaCl_2_; this inhibition was observed by the downward and rightward shifts of the dose–response curves. In addition, when exposed to ALEO, the concentration needed to elicit a 50% contraction by CaCl_2_ was greater. Other essential oils, like *Anthemis mauritiana*, also work by blocking voltage-gated Ca^2+^ channels (Karim et al., 2010). Similarly, the essential oil of *Artemisia herba-alba* (Aziz et al., 2012) blocked Ca^2+^ channels. In addition, the *in silico* prediction of binding of perillaldehyde to the active site of Ca_v_1.1 supports the hypothesis. Moreover, limonene, also present in ALEO, has also been shown to have an antispasmodic property and relax muscle via blocking of voltage-gated calcium channel openings (Carvalho et al., 2018). Furthermore, α-pinene present in ALEO formed stable associations with the Ca_v_1.1 channel via various van der Waals forces involving amino acids ILE1049, ILE1050, and PHE608 (Marghich et al., 2023).

Anticholinergic substances play a crucial role in antispasmodic effects. Therefore, carbachol was utilized for this purpose. Carbachol is a structural analog of acetylcholine that has the same contracting effect on intestinal smooth muscle by binding to M_2_ muscarinic receptors and then inhibiting adenylate cyclase or M_3_ receptors coupled to G proteins by degrading phosphatidylinositol 4,5-bisphosphate with the subsequent formation of diacylglycerol and IP_3_ (Goyal, 1988). Therefore, CCh was used to increase the basic tone of the smooth muscle of the jejunum in the rat. Exposure to ALEO caused dose-dependent spasmolytic effects on the carbachol-induced contraction of the jejunum in rats. The decrease in the contractions tonus was more pronounced for perillaldehyde compared to ALEO, with an IC_50_ value (68.59 ± 6.57 μg/mL), which was lesser by half compared to ALEO. This result allows us to assume that their antispasmodic activity might involve an action inhibiting muscarinic receptors. This hypothesis was substantiated by the observation that exposure to ALEO resulted in the dose–response curves of carbachol to be shifted down and to the right. Consequently, this inhibitory effect closely resembles to that of a noncompetitive antagonist of muscarinic receptors. These findings are consistent with the previous results, which demonstrated that *Plectranthus barbatus* exhibits non-competitive antagonism against carbachol in the smooth muscles of the ileum (Câmara et al., 2003). The *in silico* prediction of perillaldehyde interacting with muscarinic acetylcholine (M_2_ and M_3_) receptor active sites rather than Ca_v_1.1 channels is consistent with the experimental findings and confirms that ALEO and perillaldehyde act via muscarinic receptors. Although our research primarily highlights perillaldehyde’s efficacy, we acknowledge the importance of considering the minor molecules present in ALEO. To this end, we included D-limonene in our docking calculations, given its presence in ALEO at a concentration of 6.33%. Our findings indicate that D-limonene interacts with the active sites of the three targets, although its binding energies were notably lower than those of perillaldehyde. Additionally, perillaldehyde’s percentage in ALEO (91.2%) is significantly greater than that of D-limonene. These results confirm that ALEO’s activity is largely driven by perillaldehyde rather than D-limonene. Prior study has suggested that limonene contributes to reducing gastrointestinal motility (Sadraei et al., 2015), which supports its potential complementary effects in ALEO. Conversely, β-pinene and α-pinene are reported to interact less effectively with muscarinic receptors compared to atropine and other compounds (Marghich et al., 2023). Therefore, it is plausible that these minor components in ALEO may attenuate the pronounced effects observed with perillaldehyde alone. Furthermore, perillaldehyde exhibited an antibacterial effect and decreased the bacterial count by 53% (Sato et al., 2006). In addition, ALEO exhibited antioxidant and antimicrobial effects (Dahmane et al., 2017; Louail et al., 2016). All compounds exhibit absorption rates greater than 30%, with significant potential for efficient oral absorption (Pires et al. 2015). Among these, perillaldehyde stands out with a high absorption rate of 97.185% in the human intestinal tract, indicating its high potential for oral bioavailability. All those results are consistent with the strong effect of ALEO on the dysfunction of motility of the intestine. Alternatively, oils abundant in perillaldehyde could serve as flavor enhancers in food and as aromatic ingredients in perfumes, imparting a spicy essence (El-Haci et al., 2014). Perillaldehyde, which has a subtle minty scent, is derived from essential oils and declared “generally recognized safe” by the U.S. Food and Drug Administration (Tolouee et al., 2010). This classification was confirmed by results of the *in silico* toxicity assessment of perillaldehyde, which indicated a minimal acute toxic potency. This approach serves as an efficient alternative, gaining significant attention from drug developers for assessing toxicity profiles of candidate molecules (Sahota et al., 2016). Furthermore, perillaldehyde is not anticipated to cause hepatotoxicity, cancer, immunotoxicity, mutagenicity, or cytotoxicity. In addition, oral administration of mice to ALEO at dosages up to 200 mg/kg resulted in no adverse effects and no mortality. However, higher dosages of ALEO resulted in mortality, with all animals succumbing within 24 h of being given doses higher than 1,250 mg/kg, bm. The lethal dose for 50% of the population (LD_50_) of ALEO was 520–570 mg/kg, body mass (bm), ranking it in the category of slightly toxic according to the Hodge and Sterner scale. For humans, the LD_50_ value suggests that potentially risky doses could vary by approximately ±10% of this range (Mohammedi et al., 2018).

## 5 Conclusion

In summary, essential oil of *A. leucotrichus* and perillaldehyde have both relaxing and spasmolytic activities due to their ability to block muscarinic receptors and L-type voltage-gated calcium channels, with perillaldehyde preferentially binding to M_2_ and M_3_ muscarinic receptors. The present findings are consistent with those predicted by *in silico* simulations, providing a scientific basis for the traditional use of *A. leucotrichus* for digestive problems. They also open possibilities for developing a more effective and less toxic drug-utilizing perillaldehyde or synthetic molecules based on perillaldehyde.

## Data Availability

The original contributions presented in the study are included in the article/Supplementary Material; further inquiries can be directed to the corresponding authors.

## Abbreviations

ALEO, *Ammodaucus leucotrichus* essential oil; Ca_v_1.1, L-type voltage-gated calcium channel; CCh, carbachol; DMSO, dimethyl sulfoxide; EC_50_, effective concentration 50; GC/MS, gas chromatography–mass spectrometry analysis; IC_50_, half-maximal inhibitory concentration; ICCs, interstitial Cajal cells; IP_3_, inositol 1,4,5-trisphosphate; KHB, Krebs–Henseleit buffer solution; SMCs, smooth muscle cells.

## References

[B1] Abu Zarga M. H. Al-Jaber H. I. Baba Amer Z. Y. Sakhrib L. Al-Qudah M. A. Al-humaidi J. Y. G. (2013). Chemical composition, antimicrobial and antitumor activities of essential oil of *Ammodaucus leucotrichus* growing in Algeria. J. Biol. Act. Prod. Nat. 3, 224–231. 10.1080/22311866.2013.833469

[B2] Aftab T. Hakeem K. R. (2021). Medicinal and aromatic plants healthcare and industrial applications. Cham, Switzerland. 10.1007/978-3-030-58975-2

[B3] Amrani O. Marghich M. Addi M. Hano C. Chen J. T. Makrane H. (2022). The antispasmodic effect of *Warionia saharae* essential oil in experimental models and its mechanism of action. Front. Biosci. Sch. Ed. 14, 10. 10.31083/j.fbs1402010 35730435

[B4] Aziz M. Karim A. Mekhfi H. Bnouham M. Ziyyat A. Legssyer A. (2012). Antispasmodic effects of aqueous extract of *Anthemis mauritiana* maire and sennen flowers. J. Appl. Pharm. Sci. 2, 041–044. 10.7324/JAPS.2012.2908

[B5] Banerjee P. Eckert A. O. Schrey A. K. Preissner R. (2018). ProTox-II: a webserver for the prediction of toxicity of chemicals. Nucleic. Acids. Res. 46, W257–W263. 10.1093/nar/gky318 29718510 PMC6031011

[B6] Beghalia M. Ghalem S. Allali H. Belouatek A. Marouf A. (2009). Effects of an aqueous extract from *Ammodaucus leucotrichus* on calcium oxalate crystallization *in vitro* . Med. Plants 1, 37. 10.5958/j.0975-4261.1.1.006

[B7] Bellakhdar J. (2020). La pharmacopée marocaine traditionnelle: médecine arabe ancienne et savoirs populaires (2 volumes). Casablanca, Maroc: Le Fennec.

[B8] Bernard M. Fouzia D. Didler Le N. Souad F. T. Jean-Pertte R. (1997). Ammoactone, Aguaianolike from a medicinal plant, *Ammodaucus leucotrichus* . Phytochem 44, 907–910. 10.1016/S0031-9422(96)00621-8

[B9] Beyi L. Marghich M. Amrani O. Karim A. Harit T. Aziz M. (2023). Chemical composition, *in vitro* and *in silico* approaches of the relaxant effect of the jejunum using *Thymus algeriensis* Boiss. and Reut essential oil. Phytomed Plus 3, 100498. 10.1016/j.phyplu.2023.100498

[B10] Brahim A. M. S. Badr S. Mohamed G. Abderrahman A. Nadine A. Salwa E. (2014). Bioactivity and chemical quality of *Ammodaucus leucotrichus* ssp. leucotrichus Coss. \and Durieu essential oils from Morocco. Nat. Prod. Indian. J. 10, 208–214.

[B11] Câmara C. C. Nascimento N. R. F. Macêdo-Filho C. L. Almeida F. B. S. Fonteles M. C. (2003). Antispasmodic effect of the essential oil of *Plectranthus barbatus* and some major constituents on the Guinea-pig ileum. Planta Med. 69, 1080–1085. 10.1055/s-2003-45186 14750021

[B12] Carvalho P. M. M. Macêdo C. A. F. Ribeiro T. F. Silva A. A. Da Silva R. E. R. de Morais L. P. (2018). Effect of the *Lippia alba* (Mill.) N.E. Brown essential oil and its main constituents, citral and limonene, on the tracheal smooth muscle of rats. Biotechnol. Rep. 17, 31–34. 10.1016/j.btre.2017.12.002 PMC5881402 29619330

[B13] Da Costa O. B. Del Menezzi C. H. S. Benedito L. E. C. Resck I. S. Vieira R. F. Ribeiro B. H. (2014). Essential oil constituents and yields from leaves of *Blepharocalyx salicifolius* (kunt) O. Berg and *Myracrodruon urundeuva* (allemão) collected during daytime. Int. J. For. Res. 2014, 1–6. 10.1155/2014/982576

[B14] Dahmane D. Dob T. Krimat S. Nouasri A. Metidji H. Ksouri A. (2017). Chemical composition, antioxidant and antibacterial activities of the essential oils of medicinal plant *Ammodaucus leucotrichus* from Algeria. J. Essent. Oil Res. 29, 48–55. 10.1080/10412905.2016.1201015

[B15] El-Haci I. A. Bekhechi C. Atik-Bekkara F. Mazari W. Gherib M. Bighelli A. (2014). Antimicrobial activity of *Ammodaucus leucotrichus* fruit oil from Algerian Sahara. Nat. Prod. Commun. 9, 711–712. 10.1177/1934578x1400900533 25026729

[B16] Es-Safi I. Mechchate H. Amaghnouje A. Jawhari F. Z. Bari A. Cerruti P. (2020). Medicinal plants used to treat acute digestive system problems in the region of fez-meknes in Morocco: an ethnopharmacological survey. Ethnobot. Res. Appl. 20, 1–14. 10.32859/era.20.25.1-14

[B17] Fakchich J. Elachouri M. (2014). Ethnobotanical survey of medicinal plants used by people in oriental Morocco to manage various ailments. J. Ethnopharmacol. 154, 76–87. 10.1016/j.jep.2014.03.016 24685583

[B18] Goyal R. K., (1988). VI. Identification, localization and classification of muscarinic receptor subtypes in the gut. Life. Sci. 43, 2209–2220. 10.1016/0024-3205(88)90414-6 3062295

[B19] Hajib A. Danton O. Keller M. Potterat O. Bougrin K. Charrouf Z. (2023). Polyacetylenic caffeoyl amides from *Ammodaucus leucotrichus* . Phytochem 206, 113555. 10.1016/j.phytochem.2022.113555 36496003

[B20] Halla N. Heleno S. A. Costa P. Fernandes I. P. Calhelha R. C. Boucherit K. (2018). Chemical profile and bioactive properties of the essential oil isolated from *Ammodaucus leucotrichus* fruits growing in Sahara and its evaluation as a cosmeceutical ingredient. Ind. Crops. Prod. 119, 249–254. 10.1016/j.indcrop.2018.04.043

[B21] Hubatsch I. Ragnarsson E. G. E. Artursson P. (2007). Determination of drug permeability and prediction of drug absorption in Caco-2 monolayers. Nat. Protoc. 2, 2111–2119. 10.1038/nprot.2007.303 17853866

[B22] Karaki H. Urakawa N. Kutsky P. (1984). Potassium-induced contraction in smooth muscle. Res 20, 427–444. 10.1540/jsmr1965.20.427 6100197

[B23] Karim A. Berrabah M. Mekhfi H. Ziyyat A. Legssyer A. Bouali A. (2010). Effect of essential oil of *Anthemis mauritiana* Maire and Sennen flowers on intestinal smooth muscle contractility. J. Smooth. Muscle. Res 46, 65–75. 10.1540/jsmr.46.65 20383035

[B24] Laroui H. Zerargui F. Saffidine K. Guemmaz T. Trabsa H. Arrar L. (2023). Polyphenol content, antioxidant, antihemolytic and anticoagulant potentials of *Ammodaucus leucotrichus* seed extracts. Trop. J. Pharm. Res. 22, 1237–1246. 10.4314/tjpr.v22i6.13

[B25] Louail Z. Kameli A. Benabdelkader T. Bouti K. Hamza K. Krimat S. (2016). Antimicrobial and antioxidant activity of essential oil of *Ammodaucus leucotrichus* Coss. Dur. Seeds. J. Mater. Environ. Sci. 7, 2689–2695.

[B26] Maberly P. (1997). The plant book. 2nd ed. Cambridge University Press.

[B27] Makrane H. Aziz M. Mekhfi H. Ziyyat A. Legssyer A. Melhaoui A. (2019). Myorelaxant Activity of essential oil from Origanum majorana L. on rat and rabbit. J. Ethnopharmacol. 228, 40–49. 10.1016/j.jep.2018.08.036 30205180

[B28] Manssouri M. Znini M. El Harrak A. Majidi L. (2016). Antifungal activity of essential oil from the fruits of *Ammodaucus leucotrichus* Coss. and Dur., in liquid and vapour phase against postharvest phytopathogenic fungi in apples. J. Appl. Pharm. Sci. 6, 131–136. 10.7324/JAPS.2016.60520

[B29] Marghich M. Amrani O. Karim A. Harit T. Beyi L. Mekhfi H. (2023). Myorelaxant and antispasmodic effects of the essential oil of *Artemisia campestris* L., and the molecular docking of its major constituents with the muscarinic receptor and the L-type voltage-gated Ca^2+^channel. J. Ethnopharmacol. 311, 116456. 10.1016/j.jep.2023.116456 37019158

[B30] Mohammedi H. Idjeri-Mecherara S. Menaceur F. Azine K. Hassani A. (2018). Chemical compositions of extracted volatile oils of *Ammodaucus leucotrichus* L. Fruit from different geographical regions of Algeria with evaluation of its toxicity, anti-inflammatory and antimicrobial activities. J. Essent. Oil-Bearing Plants 21 (6), 1568–1584. 10.1080/0972060X.2018.1559102

[B31] Naima B. Abdelkrim R. Ouarda B. Salah N. N. Larbi B. A. M. (2019). Chemical composition, antimicrobial, antioxidant and anticancer activities of essential oil from *Ammodaucus leucotrichus* Cosson and Durieu (Apiaceae) growing in South Algeria. Bull. Chem. Soc. Ethiop. 33 (3), 541–549. 10.4314/bcse.v33i3.14

[B32] NRC (National Research Council\’s) (2011). Guide for the Care and use of laboratory animals. Eight Edition. Washighnton, United States of America: Institute for Laboratory Animal Research, The National Academeis Press.

[B33] Ozenda P. (1991). Flore et végétation du Sahara. 3rd edn. Paris: CNRS, 660.

[B35] Pires D. E. V. Blundell T. L. Ascher D. B. (2015). pkCSM: predicting small-molecule pharmacokinetic and toxicity properties using graph-based signatures. J. Med. Chem. 58, 4066–4072. 10.1021/acs.jmedchem.5b00104 25860834 PMC4434528

[B36] Rani N. Rawat D. Kaur G. Sood Y. (2022). A review on essential oil based therapies as an effective weapon against diseases. Int. J. Bot. Stud. 7 (4), 131–139.

[B37] Redouan F. Z. Benítez G. Picone R. M. Crisafulli A. Yebouk C. Bouhbal M. (2020). Traditional medicinal knowledge of Apiaceae at talassemtane national park (northern Morocco). S. Afr. J. Bot. 131, 118–130. 10.1016/j.sajb.2020.02.004

[B38] Rossum V. (1963). Cumulative dose-response curves. II. Technique for the making of dose-response curves in isolated organs and the evaluation of drug parameters. Arch. Int. Pharmacodyn. Ther. 143, 299–330. 13996119

[B39] Sadaoui N. Bec N. Barragan-Montero V. Kadri N. Cuisinier F. Larroque C. (2018). The essential oil of Algerian *Ammodaucus leucotrichus* Coss. and Dur. and its effect on the cholinesterase and monoamine oxidase activities. Fitoterapia 130, 1–5. 10.1016/j.fitote.2018.07.015 30056187

[B40] Sadraei H. Asghari G. Kasiri F. (2015). Comparison of antispasmodic effects of *Dracocephalum kotschyi* essential oil, limonene and α-terpineol. Res. Pharm. Sci. 10, 109–116. 26487887 PMC4584449

[B41] Sahota T. Danhof M. Della Pasqua O. (2016). Pharmacology-based toxicity assessment: towards quantitative risk prediction in humans. Mutagen 31, 359–374. 10.1093/mutage/gev081 26970519

[B42] Sanders K. M. (2008). Regulation of smooth muscle excitation and contraction. Neurogastroenterol. Motil. 20, 39–53. 10.1111/j.1365-2982.2008.01108.x 18402641 PMC8320329

[B43] Sanders K. M. Ördög T. Koh S. D. Torihashi S. Ward S. M. (1999). Development and plasticity of interstitial cells of Cajal. Neurogastroenterol. Motil. 11, 311–338. 10.1046/j.1365-2982.1999.00164.x 10520164

[B44] Sato K. Krist S. Buchbauer G. (2006). Antimicrobial effect of trans-cinnamaldehyde, (-)-perillaldehyde, (-)-citronellal, citral, eugenol and carvacrol on airborne microbes using an airwasher. Biol. Pharm. Bull. 29, 2292–2294. 10.1248/bpb.29.2292 17077531

[B46] Takaki M. (2003). Gut pacemaker cells: the interstitial cells of cajal (ICC). Res 39, 137–161. 10.1540/jsmr.39.137 14695026

[B47] Tolouee M. Alinezhad S. Saberi R. Eslamifar A. Zad S. J. Jaimand K. (2010). Effect of *Matricaria chamomilla* L. flower essential oil on the growth and ultrastructure of *Aspergillus niger* van Tieghem. Int. J. Food. Microbiol. 139, 127–133. 10.1016/j.ijfoodmicro.2010.03.032 20385420

[B48] Xu W. X. (2011). Advances in research on pacemaking function of interstitial cell of cajal in gastrointestinal tract. Mod. Pacemakers - Present Future 14. 10.5772/13715

[B49] Yang J. M. Chen C. C. (2004). GEMDOCK: a generic evolutionary method for molecular docking. Proteins Struct. Funct. bioinf. 55, 288–304. 10.1002/prot.20035 15048822

[B50] Ziani B. E. C. Rached W. Bachari K. Alves M. J. Calhelha R. C. Barros L. (2019). Detailed chemical composition and functional properties of *Ammodaucus leucotrichus* Cross. and Dur. and *Moringa oleifera* Lamarck. J. Funct. Foods. 53, 237–247. 10.1016/j.jff.2018.12.023

